# Ketogenic Diet Improves Brain Ischemic Tolerance and Inhibits NLRP3 Inflammasome Activation by Preventing Drp1-Mediated Mitochondrial Fission and Endoplasmic Reticulum Stress

**DOI:** 10.3389/fnmol.2018.00086

**Published:** 2018-03-20

**Authors:** Min Guo, Xun Wang, Yanxin Zhao, Qi Yang, Hongyan Ding, Qiang Dong, Xingdong Chen, Mei Cui

**Affiliations:** ^1^Department of Neurology, Huashan Hospital, Fudan University, Shanghai, China; ^2^Department of Neurology, Shanghai Tenth People’s Hospital, Tongji University, Shanghai, China; ^3^The State Key Laboratory of Genetic Engineering, Collaborative Innovativation Center for Genetics and Development, School of Life Science, Fudan University, Shanghai, China; ^4^Fudan University Taizhou Institute of Health Science, Taizhou, China

**Keywords:** ketogenic diet, β-hydroxybutyrate, mitochondrial fission, endoplasmic reticulum stress, NLRP3 inflammasome, Drp1

## Abstract

**Background**: Neuroprotective effects of ketogenic diets (KD) have been reported in stroke models, and nucleotide-binding domain (NOD)-like receptor protein 3 (NLRP3) inflammasome has also been implicated in the pathogenesis of stroke. This study aimed to investigate the effects of KD on NLRP3 inflammasome and explore the potential molecular mechanisms.

**Methods**: In *in vivo* study, mice were fed with KD for 3 weeks and then subjected to middle cerebral artery occlusion/reperfusion (MCAO/R)-injury. In *in vitro* study, SH-SY-5Y cells were treated with β-hydroxybutyrate (BHB) followed by oxygen–glucose deprivation/reoxygenation (OGD/R). NLRP3 inflammasome activation and related regulatory mechanisms were evaluated.

**Results**: Mice fed with KD had increased tolerance to MCAO/R. KD inhibited endoplasmic reticulum (ER) stress and suppressed TXNIP/NLRP3 inflammasome activation in the brain. The *in vitro* study showed BHB (10 mM) prevented the mitochondrial translocation of dynamin-related protein 1 (Drp1) to inhibit mitochondrial fission. Furthermore, BHB decreased reactive oxygen species (ROS) generation, inhibited ROS-NLRP3 pathway in OGD/R-treated cells, and suppressed ER stress-induced NLRP3 inflammasome activation.

**Conclusions**: KD may suppress ER stress and protect mitochondrial integrity by suppressing the mitochondrial translocation of Drp1 to inhibit NLRP3 inflammasome activation, thus exerting neuroprotective effects. Our findings provide evidence for the potential application of KD in the prevention of ischemic stroke.

## Introduction

Ischemic stroke, a leading cause of destructive cerebrovascular diseases, is characterized by disrupted blood flow and glucose/oxygen deprivation of brain cells, which may result in cellular dysfunction (Fisher and Saver, 2015). Current treatments for ischemic stroke are limited because of its complex molecular mechanisms (Barrett et al., 2015). Inflammation plays an important role in the pathogenesis of ischemic stroke, and the role of nucleotide-binding domain (NOD)-like receptor protein 3 (NLRP3) inflammasome in stroke has been a focus in current studies (Kawabori and Yenari, 2015). The NLRP3 inflammasome is a molecular platform in which pro-inflammatory cytokines (such as caspase-1 and interleukin-1β [IL-1β]) are activated to induce inflammation (Ogura et al., 2006). It has been confirmed that NLRP3 inflammasome is activated since the onset of ischemic stroke (Fann et al., 2013b), and treatments targeting NLRP3 inflammasome are found to be promising for the treatment of stroke (Yang et al., 2014).

Under physiological condition, NLRP3 typically localizes in the endoplasmic reticulum (ER). Upon activation, NLRP3 and its adaptor apoptosis-associated Speck-like protein (ASC) translocate to the perinuclear space, where they co-localize with ER and mitochondrial organelle clusters (Jo et al., 2016). Mitochondria are remarkably dynamic organelles that can traffic, divide and fuse. The cycles of mitochondrial fission and fusion ensure normal metabolic and bioenergetic functions (Bertholet et al., 2016). Mitochondrial fission can trigger the NLRP3 inflammasome upon RNA virus infection, in which, dynamin-related protein1 (Drp1), a key regulator of mitochondrial fission, plays an important role (Rayamajhi and Miao, 2014). Our previous study revealed that Drp1 translocating to the mitochondria mediated the mitochondrial fission, and pharmacological inhibition of Drp1 translocation prevented mitochondrial fragmentation, therefore protecting neurons from oxygen–glucose deprivation (OGD) induced injury (Zhao et al., 2013). However, the mechanism by which Drp1 regulates NLRP3-mediated inflammation in ischemic stroke remains unclear.

Following ischemia, the newly synthesized proteins accumulate to increase the unfolded protein response (UPR). Severe and prolonged UPR may induce ER stress. The ER stress sensor IRE1a can induce thioredoxin-interacting protein (TXNIP) to activate NLRP3-mediated inflammation and programmed cell death (Lerner et al., 2012). Reactive oxygen species (ROS) generated from mitochondrial fission can activate NLRP3 inflammasome to exacerbate ER stress, which further impairs the mitochondrial morphology and function (Minutoli et al., 2016). Suppression of ER stress and/or ROS generation has also been found to alleviate NLRP3-mediated inflammation in stroke.

Disruption of blood flow and/or a decrease in blood glucose concentration in ischemic stroke may cause brain injury. Ketone bodies (KBs), which include acetoacetate and β-hydroxybutyrate (BHB), may serve as alternative substrates to glucose in the brain. BHB is a major member of KBs. It is generated from fatty acid oxidation and can be converted into BHB by ketogenesis in the liver mitochondria. A ketogenic diet (KD) or BHB has been found to improve the cerebral edema and infarct volume following stroke (Suzuki et al., 2001). Moreover, BHB can prevent neuronal death induced by glucose deprivation or hypoxia (Camberos-Luna et al., 2016). Recent studies also reveal that BHB is able to suppress the NLRP3 inflammasome activation in human hepatoma HepG2 cells and monocytes (Youm et al., 2015; Bae et al., 2016). However, the role of KBs in the regulation of NLRP3 inflammasome in the central nervous system after ischemia remains unknown.

In this study, effects of KD and BHB on NLRP3 inflammasome were investigated in mice with middle cerebral artery occlusion (MCAO) and SH-SY-5Y cells subjected to OGD/reoxygenation (OGD/R), respectively, and the potential mechanism was further explored. Our results revealed that KD and BHB were able to improve cerebral ischemia by inhibiting NLRP3 inflammasome activation, which was ascribed to the suppression of Drp1-mediated mitochondrial fission and the inhibition of ER stress.

## Materials and Methods

### Animals and Dietary Protocols

Male C57BL/6 mice (4 weeks of age) were housed in the Experimental Animal Center of Fudan University at 26°C with 12 h/12 h light/dark cycles and given *ad libitum* access to food and water. Mice were randomly assigned to four groups: control group (mice were fed with standard chow [Teklab, 8664]); KD group (mice were fed with high fat, low carbohydrate diet [ResearchDiets, D12369B]); high carbohydrate (HC) diet group (mice were fed with low fat, HC diet [ResearchDiets, D12359]); CP-456773 group (mice received a single dose of oral CP-456773 [50 mg/kg, NLRP3 inflammasome inhibitor, Sigma PZ0280] for 3 weeks with standard chow). Mice were fed with assigned diets (the composition of three diets is shown in Table 1) for 3 weeks. Prior to randomization, mice in each group were fasted for 18 h to stabilize the blood glucose level and to initiate a state of ketosis. All procedures were performed in accordance with the Guide for the National Science Council of the People’s Republic of China. The study was approved by the Ethics Committee of Fudan University, Shanghai, China. The approval number from IRB is “20150572A259”. This manuscript was written in accordance with the Animal Research: Reporting *in vivo* Experiments (ARRIVE) guideline.

**Table 1:** Macronutrient composition of mouse diets used in this study.

Diet	Fat (%)*	Protein (%)*	Carb (%)*
Ketogenic diet (KD)	89.5	10.4	0.1
High carbohydrate diet (HC)	11.5	10.4	78.1
Standard lab-chow (Control)	27.5	20.0	52.6

All diets are sterilized before feeding. *Values represent percentage of total calorie.

### Establishment of the MCAO Model

Mice were anesthetized by intraperitoneal injection of ketamine at 65 mg/kg and xylazine at 6 mg/kg. A coated filament was inserted into the right middle cerebral artery (MCA) for 45-min occlusion, followed by reperfusion. The body temperature was maintained at 37°C by using a heating pad and feedback control system (Bowdoin, ME, USA) during the operation. A laser Doppler probe was placed on the skull (5 mm lateral and 2 mm posterior to the bregma) to monitor the cerebral blood flow (CBF). Mice with <25% CBF reduction after occlusion and ~80% CBF increase upon reperfusion were included for further analysis. The sham-operated mice underwent the same operation but without ischemia.

### Assessment of Infarct Volume, Neurological Deficits and Blood-Brain Barrier

Infarct volume was determined after 72-h reperfusion. Brains were harvested and sliced, followed by incubation for 1 h with 2,3,5-triphenyltetrazolium chloride (TTC). The infarct area was white, while the intact area was red. The infarct area was determined by measuring the total contralateral hemisphere and subtracting the normal tissue area on the ipsilateral hemisphere using Image Pro Plus 6.0 (Doerfler et al., 2001).

Neurological score was determined after 72-h reperfusion as follows: 0 = no deficit; 1 = forelimb weakness and torso turning to the ipsilateral side when held by tail; 2 = circling to the affected side; 3 = unable to bear weight on affected side; 4 = no spontaneous locomotor activity or barrel rolling.

Blood-brain barrier (BBB) permeability was measured after Evans Blue injection. Evans Blue (Sigma-Aldrich) was dissolved in 2% saline and injected into the right jugular vein after 72-h reperfusion (0.2 ml/kg). Evans blue was allowed to circulate in bloodstream for 2 h before mice receiving transcardially perfusion with PBS. Then, animals were sacrificed, and the brains were homogenized in 3 ml of N, N-dimethylformamide (Sigma-Aldrich), followed by incubation at 55°C for 18 h. After centrifugation at 12,000 *g* for 20 min, the concentration of Evans Blue in the supernatant was determined by measuring the absorbance at 610 nm by spectrophotometry.

Investigators blinded to grouping and treatments evaluated the infarct volume and neurological score.

### Measurement of Plasma BHB

Plasma BHB levels were detected by using an enzymatic assay kit (ColorimetricAssay Kit, cat# K632-100, BioVision Inc., Milpitas, CA, USA). On the last day of week 3, blood samples were collected from the tail of each mouse. To remove interfering substance from the serum, the sample was spun filtered (10 kDa MWCO spin filter, BioVision, cat#1997-25). Briefly, 50 μl of filtered serum was mixed with 50 μl of reaction mix (46 μl BHB assay buffer, 2 μl of BHB enzyme mix and 2 μl of substrate mix). The mixture was carefully mixed in a microtiter plate, and then incubated at room temperature for 30 min, and OD value was measured at 450 nm in a Microplate Reader (BioTek Synergy H1).

### TUNEL Staining

Animals were sacrificed after 72 h reperfusion, and brain sections (5 μm) were obtained and processed for TUNEL staining with the Apop Tag kit (Intergen, Purchase, NY, USA). Briefly, brain sections were fixed in 4% paraformaldehyde for 5 min, washed twice with PBS, and incubated in permeabilization solution (0.1% Triton X-100 and 0.1% sodium citrate) for 30 min at 70°C. Subsequently, the sections were washed twice in PBS and incubated for 60 min at 37°C in “TUNEL reaction mix”. After washing in PBS twice, sections were incubated in DAPI (Molecular Probes, Eugene, OR, USA; 1:1000) for 10 min. Finally, these sections were rinsed with distilled water and treated with anti-fade mounting medium. TUNEL-positive cells were counted from 20 fields (200×, four fields per section; BioQuant software, Oxford, UK) in each mouse.

### Cell Culture and Drug Treatment *in Vitro*

SH-SY-5Y cells (CRL-2266, ATCC) were cultured in DMEM-F12 (Sigma D8437) containing 10% fetal bovine serum (FBS) and antibiotics (100 U/ml penicillin G and 100 μg/ml streptomycin sulfate) at 37°C in a humidified atmosphere with 5% CO_2_. Twenty-four hours after seeding, differentiation was induced by lowering the FBS in culture medium to 1% with 10 μM retinoic-acid (Sigma-Aldrich, R2625) for 7 days prior to treatment. Cell morphology were evaluated under phase contrast light microscopy and culture medium was replaced once every 2 days to replenish retinoic-acid. Then at 80% confluence, cells were either treated with BHB (10 μM, OGD+BHB group) or not treated (OGD group), and then subjected to OGD/R (hypoxia [<0.1% O_2_, 95% N_2_, 5% CO_2_] and glucose-free media at 37°C for 6 h, followed by reoxygenation for 1 h). Cells in BHB group were treated with BHB (10 μM) only and cells without any treatment served as control (Ctrl group). For drug treatment, cells were primed with mitochondrial division Drp (dynamin-related GTPase) inhibitor Mdivi-1 (10 μM, Sigma M0199, OGD+MDV group), ER stress inhibitor tauroursodeoxycholic acid (TUDCA, 100 μM, Sigma T0266, OGD+TUDCA group) or ROS inhibitor N-Acetyl-L-cysteine (NAC, 5 mM, Sigma A9165, OGD+NAC group) for 30 min, and subsequently subjected to OGD/R. Specially, cells were treated with ER stress agonist tunicamycin (2 μg/ml, Sigma T7765, Tu group) for 24 h to induce ER stress prior to OGD/R.

### Lactate Dehydrogenase (LDH) and MTT Assays

Cell injury was determined by lactate dehydrogenase (LDH) assay with the CytoTox 96 NonRadioactive Cytotoxicity Assay kit (Promega, Madison, WI, USA) according to the manufacturer’s instructions. Experiment was done in triplicate. SH-SY-5Y cells were cultured in a 96-well culture plate and treated with drugs or OGD/R. At the end of the treatment, 50 μl of supernatant was transferred to each well of the 96-well plate, followed by addition of 50 μl of reconstituted substrate mix to each well. The plate was incubated at room temperature for 30 min in dark. After 50 μl of stop solution was added to each well, the absorbance was measured at 490 nm. LDH content of the conditional medium was expressed as a percentage of maximal LDH release, after the subtraction of background determined from the medium alone.

MTT assay was employed to determine the cell viability. SH-SY-5Y cells were seeded into a 96-well plate (5 × 10^3^ cells/well). In brief, 20 μl of MTT solution (5 mg/ml) was added to each well at a final concentration of 0.5 mg/mL, followed by incubation for 4 h. The supernatant was removed, and 150 μL of dimethylsulfoxide solution was added to each well, followed by incubation for 20 min. The absorbance was measured at 570 nm with a reference wavelength at 630 nm. Experiment was done in triplicate.

### Measurement of ATP

ATP generation was detected by high performance liquid chromatography (HPLC) as previously reported (Cui et al., 2010). Briefly, the culture medium was removed from the cells, and liquid nitrogen was immediately added. After evaporation on ice, 300 μL of ice-cold 0.4 M perchloric acid was added to each well. Cells were collected, and centrifuged at 14,000 *g* for 15 min at 4°C. The supernatant was neutralized with 1 M K_2_CO_3_, maintained at −80°C to precipitate the perchlorate, and then centrifuged. The supernatant was collected for HPLC. A 12-channel CoulArray 5600A (ESA Inc.) and a reverse-phase column (Lichrospher-100, Merck) were used. Signals were detected using an UV detector (#526, ESA Inc.) at 260 nm and converted by the CoulArray Analog Input Adapter prior to analysis using the CoulArray^®^ software. All peak areas were within the linear range of the standard curves.

### Measurement of Mitochondrial Membrane Potential

Mitochondrial membrane potential (MMP; Δψm) was determined by confocal microscopy after tetramethylrhodamine ethyl ester (TMRE) staining as previously described (Zhao et al., 2013). After treatment, SH-SY-5Y cells were incubated with TMRE (final concentration: 50 nM) for 20 min at 37°C. The fluorescence was detected using the BD FACS Canto™ II Flow Cytometry System (BD Biosciences, San Jose, CA, USA). Non-cellular debris and dead cells were gated out, and ~30,000 events were collected for analysis. Cells were treated with 20 μM carbonyl cyanide 4-(trifluoromethoxy) phenylhydrazone (FCCP) for 20 min to collapse Δψm, and the obtained TMRM fluorescence was used to set the threshold. Data are expressed as the percentage of cells with signal above this threshold.

### ROS Assay

SH-SY-5Y cells at 80% confluence were treated as above mentioned. Cells were then washed with PBS and incubated with ROS Fluorescent Probe-DHE (Vigorous Biotechnology, Beijing, China) for 30 min at 37°C. After washing thrice with cold PBS, florescence was measured using a microplate reader at the excitation wavelength of 485 nm and emission wavelength of 525 nm.

### Assessment of Mitochondrial Morphology

SH-SY-5Y cells were grown on poly-D-lysine-coated coverslips. Mitochondria were labeled with DsRed-Mito (1 μg per 60-mm dish, Clontech, USA) according to manufacturer’s instructions. Cells were then fixed with 4% paraformaldehyde, and coverslips were mounted using Prolong Gold Antifade Reagent with DAPI (Invitrogen). Images were captured using an inverted epifluorescence microscope (Olympus, Tokyo, Japan). Cells with a predominantly intact network of tubular mitochondria were identified as normal ones. Cells with disrupted and predominantly spherical mitochondria were identified as ones with mitochondrial fission. Mitochondrial size and shape were quantified in a blinded manner using ImageJ (NIH image).

### Drp1 Translocation

SH-SY-5Y cells were used to investigate the Drp1 translocation. Cells were cultured on poly-D-lysine-coated coverslips and transfected with 1 μg of DsRed-Mito and 1 μg of Drp1-myc. After OGD, cells were fixed and immunostained with anti-myc antibody and then with AlexaFluor 488 secondary antibody (Invitrogen). Images were captured and analyzed by confocal microscopy. About 60 cells were analyzed per group in four independent experiments.

### Mitochondrial Isolation

Mitochondria were isolated using the mitochondrial isolation kit (Pierce, Rockford, IL, USA) according to the manufacturer’s instructions. Briefly, SH-SY-5Y cells were homogenized in the Dounce homogenizer and then centrifuged at 750 *g* for 10 min at 4°C. The supernatant was further centrifuged at 12,000 *g* for 15 min at 4°C, and the pellet was then washed and retained as the mitochondrial fraction.

### Enzyme-Linked Immunosorbent Assay (ELISA) Assay

Brain tissues (the brain regions supplied by MCA) were collected after treatment and rinsed with 1× PBS. For caspase-1 activity assay, brain tissues were homogenized in lysis buffer (BioVision, Inc., Milpitas, CA, USA), followed by centrifugation at 10,000 *g* for 10 min, and then the supernatant was collected. For *in vitro* experiments, about 5 × 10^6^ cells were collected, suspended in 50 μl of pre-cold lysis buffer, incubated on ice for 10 min, and then centrifuged at 10,000 *g* for 1 min, and the supernatant was collected. Protein concentration of the supernatant was determined by Bradford assay. A Caspase-1 Fluorometric Assay kit (BioVision Inc., Milpitas, CA, USA) was used to detect the caspase-1 activity, according to the manufacturers’ instructions. The fold change in fluorescence (caspase-1 activity) as compared to that in the control group was determined.

For the measurement of IL-1β, brain tissues (the brain regions supplied by MCA) were homogenized in 20 ml of 1× PBS, and the lysate was harvested. In addition, cell culture media were also collected for the measurement. Samples were centrifuged at 5000 *g* for 5 min and the supernatant was collected. IL-1β concentration was measured using the RayBio^®^ Human IL-1 β enzyme-linked immunosorbent assay (ELISA) kit (RayBiotech, Inc., Norcross, GA, USA) according to the manufacturer’s instructions. The absorbance at 450 nm was measured immediately after stop solution was added.

### Western Blotting

The brain regions supplied by MCA were lysed with radio-immunoprecipitation assay buffer (RIPA) containing protease inhibitors (Sigma, St. Louis, MO, USA). The brain tissues were homogenized and sonicated thrice (5 s for each). All samples were centrifuged at 130,000 *g* for 15 min and the supernatant was collected. Mitochondrial proteins were obtained as described above. Proteins were separated by SDS-PAGE and then transferred to a nitrocellulose membrane which was subsequently blocked in blocking buffer (5% non-fat milk, 0.1% BSA, 0.1% Tween and 34 mmol/L NaCl, Tris, pH 7.5) for 1 h at room temperature. The membranes were subsequently incubated overnight at 4°C with following primary antibodies: anti-Drp1 (1:1000, Cell Signaling Technology, USA); anti-GRP78 (1:1000, Abcam, USA); anti-HSP70 (1:3000, Santa Cruz, USA); anti-HSP60 (1:3000, Santa Cruz); anti-p-PERK (1:2000, Cell Signaling Technology); anti-PERK (1:2000, Cell Signaling Technology); anti-p-eIF2a (1:1000, Cell Signaling Technology); anti-eIF2a (1:1000, Cell Signaling Technology); anti-ATF4 (1:1000, Cell Signaling Technology); anti-CHOP (1:2000, Thermo); anti-caspase12 (1:1000, Cell Signaling Technology); anti-NLRP3 (1:1000, Cell Signaling Technology); anti-TXNIP (1:1000, Abcam); and anti-β-actin (1:5000, Sigma-Aldrich). Secondary antibodies conjugated with horseradish peroxidase (HRP) were used, and immunoreactivity was visualized by chemiluminescence (SuperSignal Ultra, Pierce, Rockford, IL, USA) assay. Protein bands were analyzed and quantified using Scion Image.

### Statistical Analysis

Data are expressed as mean ± standard error of the mean (SEM). Comparisons were done with one-way or two-way ANOVA followed by Newman-Keuls *post hoc* testing for pair-wise comparisons. Statistical analysis was performed using SPSS version 22 (IBM, Armonk, NY, USA). A value of *P* < 0.05 was considered statistically significant.

## Results

### KD Enhanced Ischemic Tolerance in MCAO Mice

**Figure 1:**
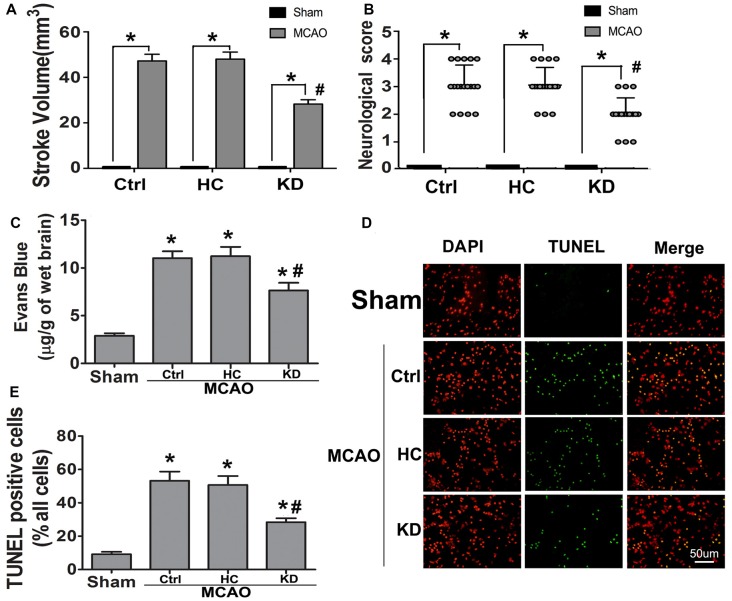
Ketogenic diets (KD) improved ischemic tolerance in middle cerebral artery occlusion (MCAO) mice. Sham-operated and MCAO mice were fed with different diet protocols, and infarct volume (**A**, *n* = 6 per group), neurological score (**B**, *n* = 18 per group), and blood-brain barrier (BBB) permeability (**C**, *n* = 6 per group) were assessed. **(D)** Fewer TUNEL-positive cells were observed in KD-fed MCAO mice (Scale bar: 50 μm). Brain sections were subjected to TUNEL staining and positive cells were counted (nuclei: DAPI). **(E)** Quantification of TUNEL-positive cells in different groups (*n* = 6 per group). Data are shown as mean ± standard error of the mean (SEM); *P < 0.01 vs. sham-operated mice, ^#^*P* < 0.05 vs. MCAO group.

Following 3-week KD treatment, the daily food intake and calories intake of KD group gradually decreased while they were gradually increased in HC group Supplementary Figures S1C,E). However, due to the high-calorie content of KD, there was no difference in total calories intake among the three groups during the 3 weeks (Supplementary Figure S1D). The body weight decreased in the KD group, but the BHB level in the KD group was over four times than that in the HC and control groups (Supplementary Figures S1A,B), which was consistent with previously reported (Su et al., 2000; Thio et al., 2006). To assess whether KD improved the ischemic tolerance, infarct volume, neurological score, and BBB permeability were determined in three groups. Results showed KD significantly reduced infarct area and neurological score in MCAO mice (Figures 1A,B). BBB permeability in the KD group was reduced by 30% as compared to the control and HC groups (Figure 1C). Then, the effect of KD on ischemia-induced cellular apoptosis was further investigated. As shown in Figures 1D,E, fewer TUNEL-positive cells were observed in the KD group as compared to the control or HC groups. These findings indicate KD improves ischemic tolerance in MCAO mice.

### KD Decreased TXNIP/NLRP3 Inflammasome Induction After Brain Ischemia

**Figure 2:**
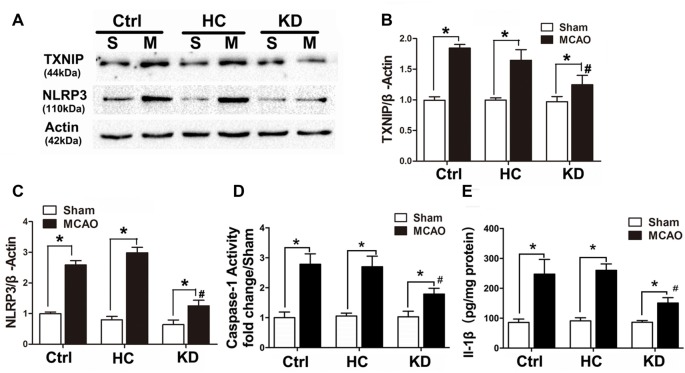
KD suppressed thioredoxin-interacting protein (TXNIP)-nucleotide-binding domain (NOD)-like receptor protein 3 (NLRP3) inflammasome pathway in MCAO mice. Sham-operated and MCAO mice (*n* = 6 per group) were fed with different diet protocols and protein expression was detected by Western blotting. **(A)** Representative blots from six independent experiments with similar results are shown. TXNIP **(B)** and NLRP3 **(C)** protein expression were determined. **(D)** Caspase-1 activity and IL-1β concentration **(E)** were determined by enzyme-linked immunosorbent assay (ELISA). Caspase-1 activity was detected on brain lysates and compared to that in sham-operated mice. IL-1β concentration is expressed per mg of total protein in brain tissue lysates. Data are shown as mean ± SEM; *P < 0.01 vs. sham-operated mice, ^#^*P* < 0.05 vs. MCAO group.

**Figure 3:**
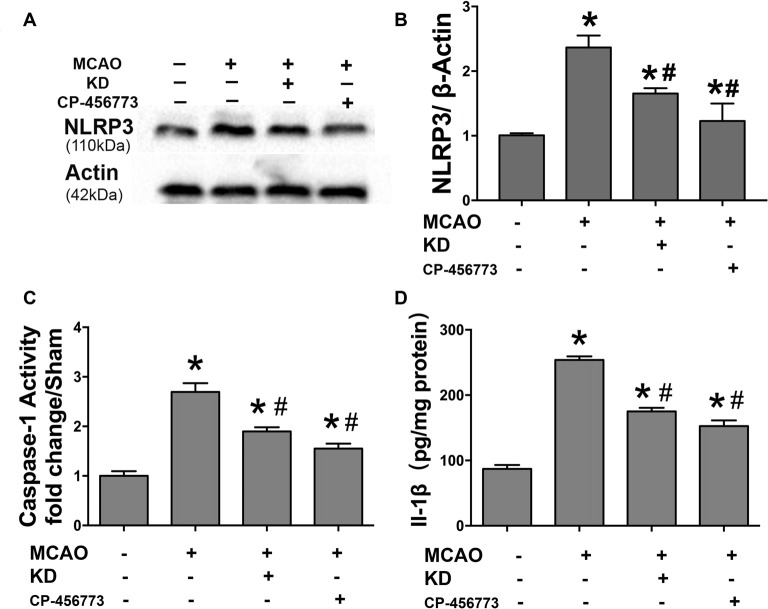
NLRP3 inflammasome inhibitor CP-456773 suppressed NLRP3 mediated inflammation response in MCAO mice. **(A,B)** Sham-operated and MCAO mice (*n* = 6 per group) were fed with different diet protocols and the protein expression of NLRP3 was detected by Western blotting. Representative blots from six independent experiments with similar results are shown. Caspase-1 activity **(C)** and IL-1β concentration **(D)** were detected by ELISA. Caspase-1 activity was determined on brain lysates and compared to that in sham-operated mice. IL-1β production is expressed per mg of total protein in brain tissue lysates. Data are shown as mean ± SEM; *P < 0.01 vs. sham-operated mice, ^#^*P* < 0.05 vs. MCAO group.

Neuroinflammation plays a significant role in neuronal and glial cell death during ischemic stroke (Seifert and Pennypacker, 2014). The NLRP3 inflammasome is crucial to this inflammatory response, and TXNIP is essential for NLRP3 inflammasome activation. Thus, the TXNIP-NLRP3 pathway was further investigated in MCAO mice. As shown in Figure 2, TXNIP and NLRP3 expression increased in the brain of MCAO mice, which was accompanied by elevated caspase-1 activity and IL-1β concentration. However, these elevations were inhibited by KD. In mice treated with NLRP3 inflammasome inhibitor CP-456773, NLRP3 inflammasome activation was significantly suppressed, and the caspase-1 activity and IL-1β secretion were also reduced markedly (Figure 3). This protective effect was further investigated *in vitro* using primary neurons treated with BHB, and results showed BHB suppressed TXNIP/NLRP3 activation (Supplementary Figure S2). These findings indicate that KD decreases NLRP3 inflammasome activation after brain ischemia.

### BHB Improved Cell Viability and Prevented Mitochondrial Fission in Cells Subjected to OGD/R

**Figure 4:**
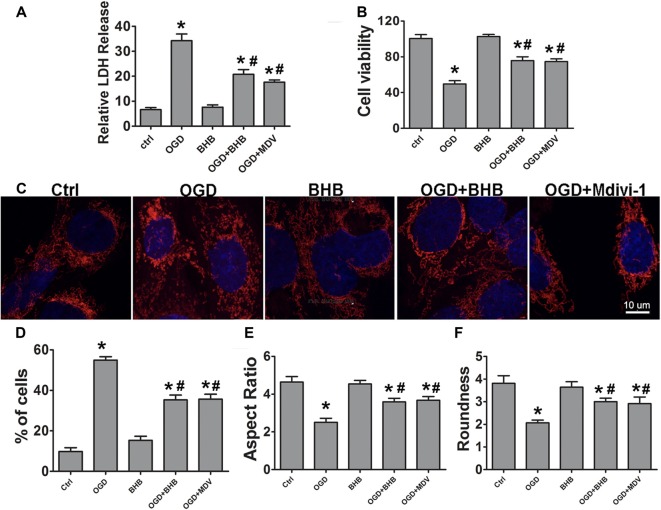
β-hydroxybutyrate (BHB) and mdivi-1 influenced cell survival and mitochondrial morphology in response to oxygen–glucose deprivation/reoxygenation (OGD/R) damage. BHB and mdivi-1 treatment inhibited lactate dehydrogenase (LDH) release **(A)** and improved cell viability **(B)** after OGD/R. Data were from six independent experiments. **(C)** Cultured SH-SY-5Y cells were labeled with MitoDs-red for the evaluation of mitochondrial morphology. Scale bar: 10 μm **(D)** Percentage of cells with truncated or fragmented mitochondria. Quantification was performed in a blind manner from more than 200 cells in at least 10 randomly selected fields at 200× from three independent experiments. **(E,F)** Quantification of mitochondrial morphology. Roundness was calculated as perimeter^2^/4π area. Aspect ratio is a measurement of major/minor axes. Both of these values approach 1 as the particle becomes more circular. Quantification was performed from 10 to 15 randomly selected cells in three independent experiments. Data are shown as mean ± SEM; *P < 0.01 vs. Ctrl group, ^#^*P* < 0.05 vs. OGD group.

The effects of BHB on ischemia-related injury were further investigated in an OGD/R cell model. BHB treatment prior to OGD/R increased cell viability and decreased LDH release after OGD/R (Figures 4A,B). Under OGD/R, mitochondrial dynamics towards fission play crucial roles in the NLRP3 inflammasome activation and apoptosis induction (Otera and Mihara, 2012). The balance between mitochondrial fission and fusion controls the mitochondrial morphology and function (Westermann, 2002). Thus, the mitochondrial morphology was investigated in SH-SY-5Y cells. Results showed OGD/R caused significant mitochondrial fragmentation, whereas BHB prevented the mitochondrial fission (Figure 4C). Moreover, a higher proportion of smaller, rounder mitochondria was present in cells after OGD/R. Consistent with this finding, cells treated with BHB displayed a higher proportion of tubular, long mitochondria (Figures 4D–F). Because mitochondrial fission was mainly controlled by Drp1, cells were treated with the small molecule mitochondrial division inhibitor (mdivi-1) to inhibit Drp1. These cells showed decreased mitochondrial fragmentation. These findings reveal OGD/R likely causes excessive mitochondrial fission, and BHB may induce the mitochondrial morphological changes by inhibiting Drp1-mediated mitochondrial fission.

### BHB Prevented Drp1 Mitochondrial Translocation in Cells Subjected to OGD/R

**Figure 5:**
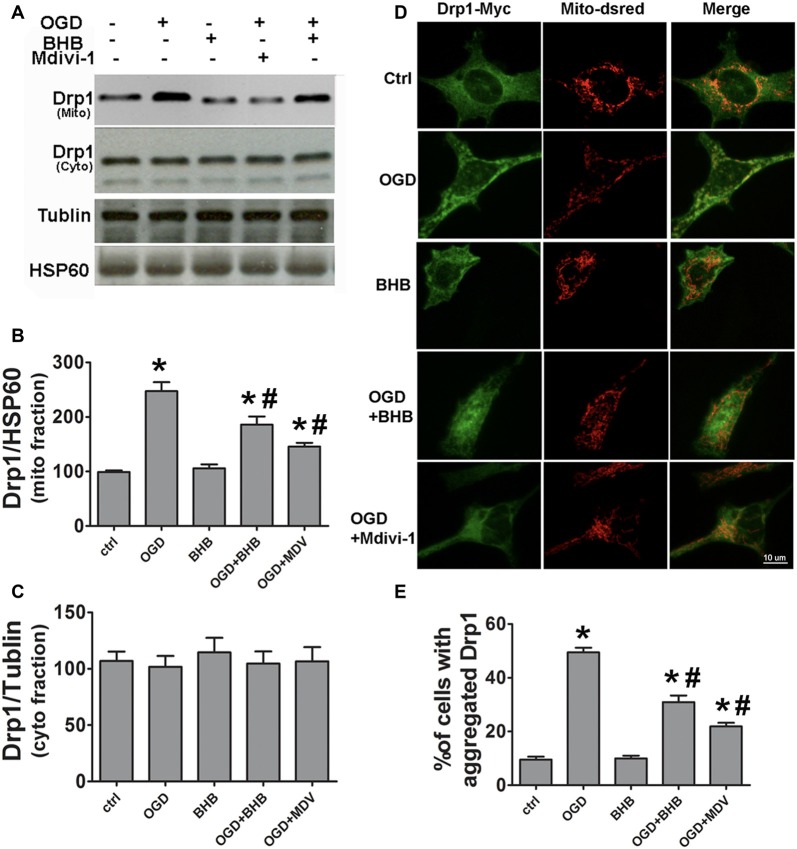
BHB and mdivi-1 prevented dynamin-related protein 1 (Drp1) mitochondrial translocation after OGD/R. **(A–C)** Protein expression of mitochondrial fission protein Drp1 in the mitochondria and cytoplasm was detected by Western blotting. Representative blots from six independent experiments with similar results are shown. **(D)** SH-SY-5Y cells were co-transfected with DsRed-Mito and Drp1-myc. Scale bar: 10 μm. **(E)** Quantification of aggregated Drp1 in cells subjected to different treatments. Data are shown as mean ± SEM; *P < 0.01 vs. Ctrl group, ^#^*P* < 0.05 vs. OGD group.

SH-SY-5Y cells were used to further investigate the Drp1 mitochondrial translocation. Mitochondria were isolated and Drp1 protein expression was determined. Mitochondrial Drp1 expression increased after OGD/R, but it was reversed by BHB pre-treatment (Figures 5A–C). Drp1 is predominantly cytosolic, and only about 3% of Drp1 localizes in the mitochondria (Smirnova et al., 2001). Our results indicated Drp1 translocated from the cytosol to the mitochondria after OGD/R. Furthermore, cells were co-transfected with Drp1-myc and DsRed-Mito. OGD/R induced Drp1 aggregation and colocalization with the mitochondria, but BHB significantly reduced Drp1 mitochondrial translocation, similar to the findings after mdivi-1 pre-treatment (Figures 5D,E).

### BHB Improved Mitochondrial Function and Suppressed NLRP3 Inflammasome Activation by Reducing ROS Production in Cells Subjected to OGD/R

**Figure 6:**
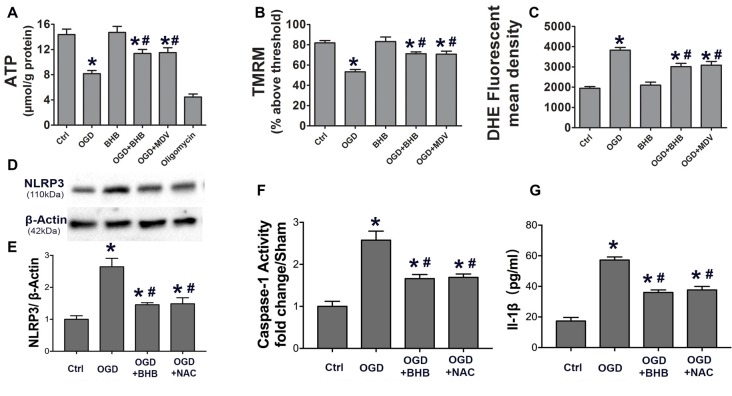
BHB and mdivi-1 prevented mitochondrial function and reactive oxygen species (ROS) inhibitor N-acetyl-L-cysteine (NAC) suppressed NLRP3 inflammasome after OGD/R. **(A)** BHB and mdivi-1 reversed the decreased ATP production after OGD/R. Oligomycin (oligo, 10 μM, a complex V inhibitor), as a positive control, significantly reduced ATP level. Data were normalized to those from oligomycin-treated cells. BHB and mdivi-1 reversed the increase in ROS production **(C)** and the decrease in mitochondrial membrane potential (MMP) **(B)** after OGD/R. **(D,E)** The ROS inhibitor NAC was added before OGD, and the protein expression of NLRP3 was detected by Western blotting. Representative blots from six independent experiments with similar results are shown. **(F)** Caspase-1 activity was detected on cell lysates and compared to that in sham-operated mice. **(G)** IL-1β production in culture media. Data are shown as mean ± SEM; *P < 0.01 vs. Ctrl group, ^#^*P* < 0.05 vs. OGD group.

To determine whether the maintenance of mitochondrial function is one of the mechanisms by which BHB exerts a protective effect on OGD/R, the ATP level in SH-SY-5Y cells was detected by HPLC, and a fluorescent cationic dye was used to measure the Δψm. Oligomycin, an inhibitor of the electron transport chain complex V, was used as a positive control for ATP measurement. As expected, OGD/R significantly reduced the ATP level, which, however, was partially reversed by BHB pre-treatment (Figure 6A). Moreover, BHB conferred protection against Δψm reduction induced by OGD/R (Figure 6B).

Because excessive ROS are generated in dysfunctional mitochondria, the effect of BHB on ROS production was further investigated. BHB pre-treatment decreased ROS production (Figure 6C). ROS is a strong NLRP3 inflammasome activator. Our results showed BHB and ROS inhibitor NAC (NAC) pre-treatment attenuated NLRP3 expression as well as reduced caspase-1 activity and IL-1β release (Figures 6D–G) after OGD/R. These results indicate that BHB suppresses NLRP3 activation by inhibiting ROS production in cells subjected to OGD/R.

### KD Attenuated ER Stress After Brain Ischemia

**Figure 7:**
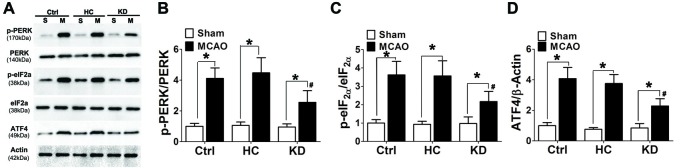
KD suppressed the p-PERK-p-eIF2α-ATF4 pathway in MCAO mice. Sham-operated and MCAO mice (*n* = 6 per group) were fed with different diet protocols and Western blotting **(A)** was used to determine the protein expression of p-PERK/PERK **(B)**, p-eIF2a/eIF2a **(C)** and ATF4/β-actin **(D)**. Representative blots from six independent experiments with similar results are shown. Data are shown as mean ± SEM; *P < 0.01 vs. sham-operated mice, ^#^*P* < 0.05 vs. MCAO group.

Ischemia and hypoxia commonly cause ER stress due to the reduced protein folding capacity of ER, thus leading to the accumulation of misfolded proteins. ER stress involves three main signaling pathways: PERK-eIF2-ATF4, IRE1-XBP1 and ATF6 pathways (Xin et al., 2014). The protein expression of p-PERK, p-eIF2α and ATF4 was detected by Western blotting in this study. Results showed ischemia significantly increased the protein expression of p-PERK, p-eIF2α and ATF4 in the brain of MCAO mice. However, the activation of p-PERK-p-eIF2α-ATF4 pathway was inhibited in the KD group (Figure 7).

**Figure 8:**
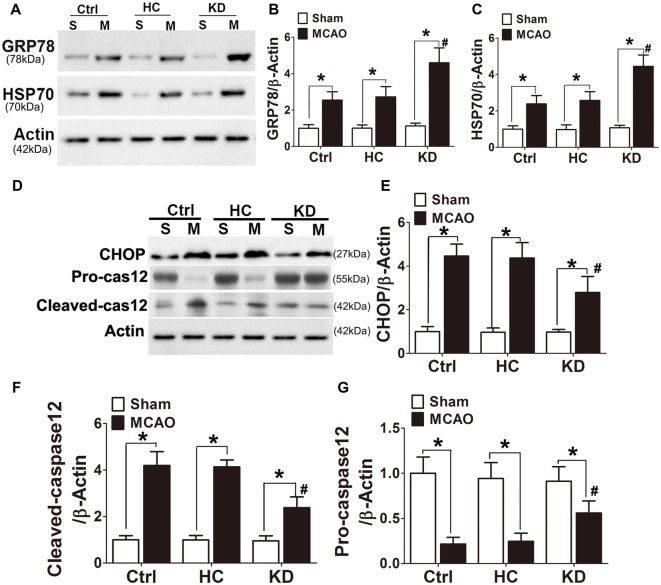
KD increased GRP78/HSP70 level but attenuated CHOP and cleaved caspase 12 expression in MCAO mice. Sham-operated and MCAO mice (*n* = 6 in each group) were fed with different diet protocols and Western blotting **(A,D)** was performed to determine the protein expression of GRP78 **(B)**, HSP70 **(C)**, CHOP **(E)**, cleaved-caspase 12 **(F)** and pro-caspase 12 **(G)**. Representative blots from six independent experiments with similar results are shown. Data are shown as mean ± SEM; *P < 0.01 vs. sham-operated mice, ^#^*P* < 0.05 vs. MCAO group.

GRP78, a chaperone protein in ER stress, can inhibit protein misfolding and exert negative regulatory effects on ER stress. HSP70 is an important chaperone regulated by ER stress. In this study, the GRP78 and HSP70 expression was higher in the KD group (Figures 8A–C) than in other two groups. CHOP and cleaved caspase-12 are ER-specific apoptotic promoters. The expression of both CHOP and cleaved caspase-12 markedly reduced, and pro-caspase-12 protein expression increased in KD-treated mice with MCAO (Figures 8D–G). These findings indicate that ER stress is attenuated in KD-treated mice with MCAO.

### BHB Suppressed NLRP3 Inflammasome Activation by Down-Regulating ER Stress in Cells Subjected to OGD/R

**Figure 9:**
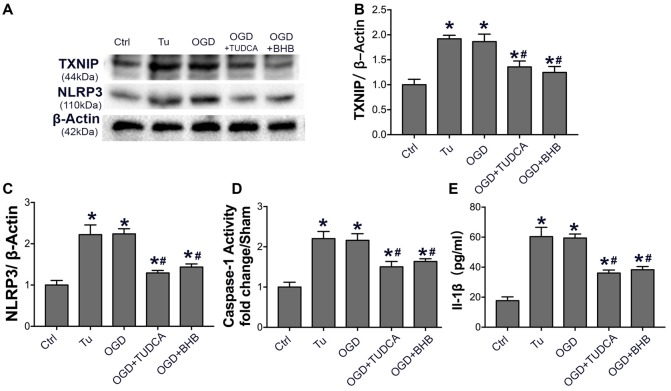
BHB and endoplasmic reticulum (ER) stress inhibitor tauroursodeoxycholic acid (TUDCA) suppressed NLRP3 inflammasome after OGD/R. **(A–C)** Protein expression of TXNIP and NLRP3 was detected by Western blotting. Representative blots from six independent experiments with similar results are shown. **(D)** Caspase-1 activity was detected on cell lysates and compared to that in sham-operated mice. **(E)** IL-1β concentration of culture medium. Data are shown as mean ± SEM; *P < 0.01 vs. Ctrl group, ^#^*P* < 0.05 vs. OGD group. Tu, tunicamycin.

Because ER stress can activate the NLRP3 inflammasome (Menu et al., 2012), the NLRP3 expression, IL-1β content and caspase-1 activity were further detected in SH-SY-5Y cells subjected to OGD/R. Our results showed OGD triggered NLRP3 activation, as observed after the use of ER stress agonist tunicamycin. However, BHB pre-treatment significantly inhibited NLRP3 expression, IL-1β release, and caspase-1 activity. Further, the ER stress TUDCA also suppressed NLRP3 activation *in vitro* (Figure 9), indicating that BHB is able to suppress NLRP3 inflammasome activation.

## Discussion

Inflammation-mediated cell dysfunction is an initial event in the ischemic stroke, and contributes to the development of neurological deficits (Tobin et al., 2014). The results of the present study showed that, under ischemic conditions, cellular NLRP3 inflammasome was activated by hypoxia-ischemia insult, thus leading to brain tissue damage. Moreover, KD pre-treatment suppressed NLRP3 inflammasome activation by inhibiting Drp1-mediated mitochondrial fission and ER stress, which alleviated brain dysfunction in ischemic stroke.

KD is a high-fat diet in which carbohydrates are nearly eliminated, and it provides sufficient protein for growth but insufficient amounts of carbohydrates for the body’s metabolic needs. Thus, the oxidation of a large amount of fat in liver mitochondria leads to elevated circulating KBs (acetoacetate, β-hydroxybutyrate and acetone; Gasior et al., 2006). Eventually, KBs in the circulation readily cross the BBB and are consumed as an alternative fuel in the brain under some conditions such as fasting, extensive physical exercise and KD (Hartman et al., 2007). In fact, the capability of brain to consume KBs is considered as a form of cerebral metabolic adaptation (Prins, 2008).

KD, which has been successfully used to treat drug-resistant epilepsy, has been found to confer neuroprotective effects on the cerebral ischemic injuries (Shaafi et al., 2014). Actually, in animal studies, a ketogenic state (induced by KD, exogenously administered KBs or calorie restriction) is proven to exert an overall significant protective effect on outcomes (decreased lesion volume, neurological score and edema, and improved survival, neuronal count and behavior) following brain ischemia in various models, including the MCAO model, vessel occlusion model and cardia arrest model (Gibson et al., 2012). Both chronic interventions like calorie restriction for 3 months or KD for 3 weeks and acute interventions like calorie restriction for 4 days or ketone administration from several days to 30 min before the onset of experimental stroke can exert protective effects (Suzuki et al., 2001; Puchowicz et al., 2008; Tai et al., 2008; Yoon et al., 2011). Moreover, it is also beneficial for the adoption to calorie restriction 1 h after ischemia and supplement with exogenous ketones immediately or 30 min after ischemic event (Suzuki et al., 2002; McEwen and Paterson, 2010). In the present study, our results also indicate that 3-week KD was able to enhance the brain ischemic tolerance to MCAO in mice, as demonstrated by improvements of infarct area, neurological score, BBB permeability and cellular apoptosis.

In spite of emerging evidence from preclinical investigations demonstrating the beneficial effects of KD, mechanisms underlying its effects are not well studied. Limited evidence from precious studies suggest that KD is able to alleviate excitotocity, oxidative stress and apoptosis (Shaafi et al., 2014). Puchowicz et al. (2008) found the hypoxia inducible factor 1α (HIF-1α) level was elevated after 3-week KD treatment because increased succinate inhibited the prolyl-hydroxylase, an enzyme responsible for the degradation of HIF. Our previous study reported the transcription of HIF target genes including erythropoietin (EPO), endothelial cell growth factor (VEGF), Glut-1 and MCT4 increased and this increase was partly related to the elevated HIF expression (Yang et al., 2017). Moreover, Ly-6CLo monocytes and/or macrophages express hydroxy-carboxylic acid receptor 2 (HCA2), a receptor for BHB, which has been reported to reduce neuroinflammation and exert a protective effect on the brain injury after KD (Rahman et al., 2014; Offermanns and Schwaninger, 2015). Other inflammatory responses, especially NLRP3 inflammasome, are also important in post-ischemic damage. Nevertheless, the effects of KD on NLRP3 inflammasome in the central nerves system remain unclear.

The NLRP3 inflammasome is an important component in innate immune response, and contributes significantly to ischemic brain injury following stroke. The NLRP3 inflammasome is activated following cerebral ischemia, and pharmacological inhibition of NLRP3-mediated inflammatory response can prevent the deterioration of cerebral function (Fann et al., 2013a,b). In this study, whether the neuroprotective effects of KD pre-treatment were related to the inhibition of NLRP3-mediated inflammatory response was further investigated. Our results revealed that MCAO and OGD induced TXNIP/NLRP3 inflammasome activation and significantly increased caspase-1 activity and IL-1β release. However, KD or BHB pre-treatment significantly suppressed the NLRP3-mediated inflammatory response, as confirmed after use of the NLRP3 inhibitor CP456773. However, the specific mechanism by which KD regulates NLRP3 inflammasome remains still unclear.

Although increasing evidence suggests that mitochondria serve as a regulator of NLRP3 inflammasome activation by producing mitochondrial ROS or DNA in response to damage (Zhou et al., 2011), the role of mitochondrial dynamics in the inflammasome pathway in neurons is still unknown. Our results indicated that Drp1 acted a downstream factor regulated by KD and could regulate the NLRP3 inflammasome activation during ischemic injury. Drp1, normally found in the cytosol, is a GTPase that mediates mitochondrial fission (Archer, 2013). Phosphorylated Drp1 can translocate to the mitochondrial outer membrane, where it interacts with the mitochondrial fission protein Fis1 to induce mitochondrial fission, thus generating ROS and activating NLRP3 inflammasome (Knott et al., 2008; Archer, 2013). Our results showed OGD/R induced Drp1 mitochondrial translocation to cause aberrant mitochondrial fission, thus leading to the significant NLRP3 inflammasome activation. In contrast, BHB pre-treatment suppressed Drp1 translocation and mitochondrial fragmentation, attenuating NLRP3 inflammasome activation. These results were consistent with previously reported that BHB could block the NLRP3 inflammasome activation (Youm et al., 2015). In addition, abnormal mitochondrial function, including decreased ATP generation and MMP, could be reversed by BHB as well as Drp1 inhibitor mdivi-1. Taken together, our results provide a molecular insight into the effects of BHB on the mitochondrial dynamics-regulated NLRP3 inflammasome activation.

It has been proposed that increased ROS due to mitochondrial dysfunction can be sensed by the complex TXNIP and then induce the dissociation of this complex (Gross et al., 2011). In normal cells, TXNIP is constitutively maintained in the reduced state; in case of increased ROS production, this complex dissociates and TXNIP binds to the LRR region of NLRP3, leading to the NLRP3 inflammasome activation (Tschopp and Schroder, 2010). In the present study, BHB pre-treatment reduced ROS generation in cells subjected to OGD/R, and BHB or ROS inhibitor NAC could attenuate the activation of TXNIP/NLRP3 inflammasome, indicating that BHB may protect cells against ROS-mediated NLRP3 activation following OGD/R.

ER stress is an essential progress in the brain ischemia-reperfusion injury (Roussel et al., 2013). The ER stress sensors PERK and IRE1α can induce TXNIP to activate the NLRP3 inflammasome (Lerner et al., 2012; Oslowski et al., 2012). IRE1α also induces ROS-dependent NLRP3 translocation to mitochondria, leading to the release of mitochondrial signals that further activate the inflammasome (Bronner et al., 2015). As expected, the p-PERK-p-eIF2α-ATF4 pathway was activated and CHOP expression increased significantly in the brain of MCAO mice, suggesting ER stress, which was significantly attenuated in the KD group. *In vivo*, the ER stress inducer tunicamycin or OGD/R activated the NLRP3 inflammasome, but BHB pre-treatment reduced this inflammation response, which was similar to the effects of ER stress inhibitor TUDCA. Collectively, these results indicate that KD or BHB is able to ameliorate ER stress-associated NLRP3 inflammasome activation.

When considering KD as a preventive therapy for high risk population of stroke, we must take into account the clinical safety, especially if the high cholesterol content of KD may cause dyslipidemia and increase the risk for stroke. It was reported that the postprandial triacylglycerol (TG) reduced, oxidized low density lipoprotein (LDL) remained unchanged and the high-density lipoprotein increased after 6-week KD treatment in normal weight men (Sharman et al., 2002). Furthermore, KD shifts the LDL particle distribution to a larger size, resulting in significant increases in peak and mean LDL diameter and decrease in the proportion of small, dense LDL particles (Meckling et al., 2002; Sharman et al., 2002; Dashti et al., 2003; Volek et al., 2005), which is considered beneficial because small LDL particles are atherogenic. In fact, the majority of recent studies demonstrate that physiological ketosis can actually benefit the blood lipid profiles (Brehm et al., 2003; Shai et al., 2008; Volek et al., 2009).

There were several limitations in this study. First, in the *in vitro* study, BHB was used to mimic the KD in the *in vivo* study. KD was adopted in animals in order to achieve a prolonged neuroprotective state before ischemia. Although BHB is the major component of KBs and the most widely used ingredient *in vitro*, the possibility that the mice are also protected by components other than BHB can not be excluded, because KD has multiple ingredients. Second, we did not assess the effects of other KBs like acetoacetate* in vitro*, thus whether other KBs work in the same way as BHB remains still unclear. Further studies are needed to elucidate these issues.

## Conclusion

**Figure 10:**
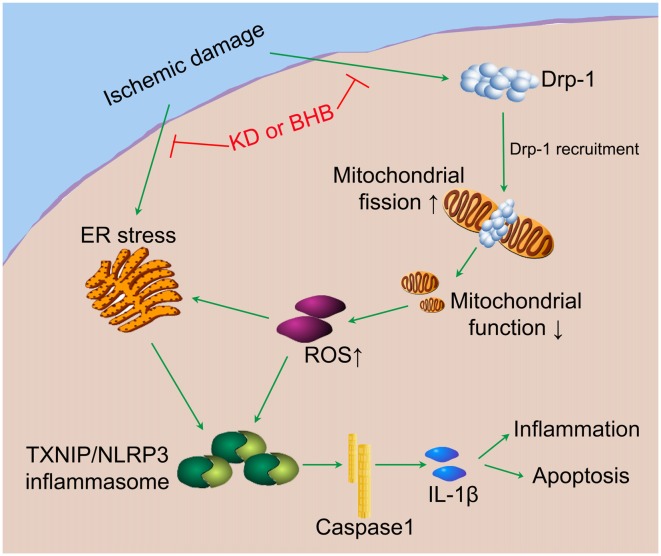
The potential mechanistic pathway by which KD or BHB regulates NLRP3 inflammasome activation. Ischemic damage causes Drp1-mediated mitochondrial fission and ER stress. Excessive ROS production aggravates ER stress and induces TXNIP/NLRP3 activation. The resultant maturation of IL-1β is responsible for the inflammation and cell apoptosis, leading to the central nervous system dysfunction. KD or BHB suppresses Drp1 recruitment and ER stress, thereby alleviating inflammation and improving brain ischemic injury.

Our results indicate that KD or BHB can prevent NLRP3 inflammasome activation and thereby improve the brain ischemic tolerance. As summarized in Figure 10, KD or BHB may inhibit Drp1 mitochondrial translocation to protect mitochondrial morphology and function, which prevents ROS-associated NLRP3 inflammasome activation. Further, BHB treatment may also ameliorate ER stress-associated NLRP3 inflammasome activation. Although prophylactic KD therapy is still not used in clinical settings, our findings confirm the neuroprotective effects of KD and provide evidence on the potential molecular mechanism underlying these effects, which support the potential clinical utility of KD as nutritional supply in acute cerebrovascular diseases.

## Availability of Data and Materials

All data in this study are included in this published article and its supplementary documents.

## Author Contributions

MG and XW: contributed equally to this work. XC and MC: co-correspondence for this article. MC, YZ and QD: conceived and designed the experiments. MG, XW and QY: performed the experiments. MG, QD and HD: analyzed the data. XC, QY, HD and YZ: contributed reagents/materials/analysis tools. MG and MC: drafted the article.

## Conflict of Interest Statement

The authors declare that the research was conducted in the absence of any commercial or financial relationships that could be construed as a potential conflict of interest.

## Abbreviations

BBB: blood-brain barrier
BHB: b-hydroxybutyrate
Drp1: dynamin-related protein 1
IL-1β: interleukin-1β
KBs: ketone bodies
KD: ketogenic diets
MCAO/R: middle cerebral artery occlusion/reperfusion
MMP: mitochondrial membrane potential
NLRP3: nucleotide-binding domain (NOD)-like receptor protein 3
OGD/R: oxygen–glucose deprivation/reoxygenation
ROS: reactive oxygen species
TTC: 2,3,5-triphenyltetrazolium chloride
TXNIP: thioredoxin-interacting protein
UPR: unfolded protein response.
