# Supplementary material for: Ketogenic Diet Improves Brain Ischemic Tolerance and Inhibits NLRP3 Inflammasome Activation by Preventing Drp1-Mediated Mitochondrial Fission and Endoplasmic Reticulum Stress
Source: Front Mol Neurosci. 2018 Mar 20;11:86. doi: 10.3389/fnmol.2018.00086 (PMC5890101; doi:10.3389/fnmol.2018.00086)
Supplement: Supplementary file 1 [file Image_1.pdf]

Ketogenic diet improves brain ischemic tolerance and inhibits NLRP3 inflammasome activation by preventing Drp1-mediated mitochondrial fission and endoplasmic reticulum stress

Running title: ketogenic diet inhibits NLRP3 inflammasome

Min Guo[1#], Xun Wang[1#], Yanxin Zhao[2], Qi Yang[1], Hongyan Ding[1], Qiang Dong[1], Xindong Chen[3*], Mei Cui[1*]
1 Department of Neurology, Huashan hospital, Fudan University, Shanghai, China
2 Department of Neurology, The 10th People's Hospital, Tongji University, Shanghai, China
3 The State Key Laboratory of Genetic Engineering, Collaborative Innovativation Center for Genetics and Development, School of Life Science, Fudan University, Shanghai, China
Fudan University Taizhou Institute of Health Science, Taizhou, China

[#] Min Guo and Xun Wang contributed equally to this work.

Min Guo: Department of Neurology, Huashan hospital, Fudan University, Shanghai, China
Telephone Number: 021-52888160
E-mail: qylcguomin@163.com
Fax Numbers: 62481088
Xun Wang: Department of Neurology, Huashan hospital, Fudan University, Shanghai, China
Telephone Number: 021-52888164
E-mail: 08301010222@fudan.edu.cn
Fax Numbers: 62481088
Yanxin Zhao: Department of Neurology, The 10th People's Hospital, Tongji University, Shanghai, China
Telephone Number: 52888164
E-mail: zhao_yanxin@126.com
Fax Numbers: 62481088
Qi Yang: Department of Neurology, Huashan hospital, Fudan University, Shanghai, China
Telephone Number: 021-52887145
E-mail: yang_qi@outlook.com
Fax Numbers: 62481088
Hongyan Ding: Department of Neurology, Huashan hospital, Fudan University, Shanghai, China Telephone Number: 021-52887145
E-mail: hyding2001@163.com
Fax Numbers: 62481088
Qiang Dong: Department of Neurology, Huashan hospital, Fudan University, Shanghai, China
Telephone Number: 021-52887145
E-mails: dong_qiang@fudan.edu.cn ;
Fax Numbers: 62481088
Correspondence:

Xingdong Chen, The State Key Laboratory of Genetic Engineering, Collaborative Innovativation Center for Genetics and Development, School of Life Science, Fudan University, Shanghai, China.
Fudan University Taizhou Institute of Health Science, Taizhou, China
No. 2005 Songhu Road, Shanghai 200433, China
E-mail: xingdongchen@fudan.edu.cn
Mei Cui, Department of Neurology, Huashan hospital, Fudan University, No.12 Middle Wulumuqi Road, Shanghai 200040, China
E-mail: cuimei@fudan.edu.cn
Telephone Number: 021-52887145

Fig S1

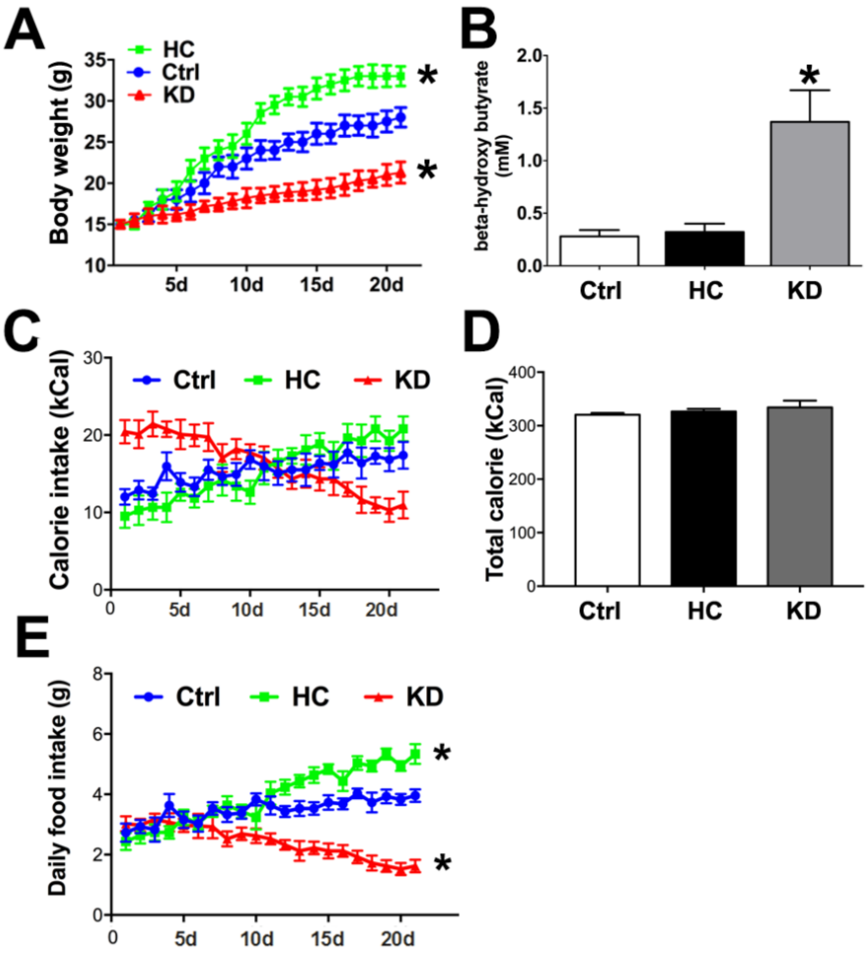

Fig S1. The body weight (A), serum β-hydroxybutyrate level (B), daily caloric intake (C) , total caloric intake (D) and daily food intake(E) in different groups. Data are shown as mean ± SEM (n = 6 per group). *P<0.05 *vs* Ctrl group. HC: high carbohydrate diet; KD: ketogenic diet; Ctrl: standard diet.

Fig S2

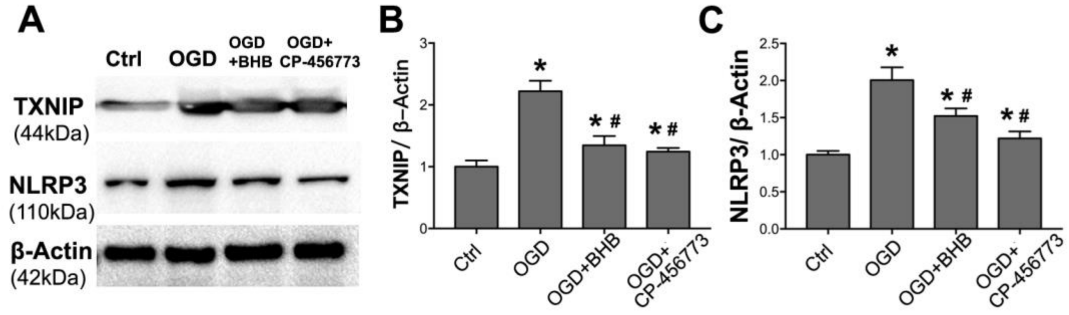

Fig S2. BHB suppressed NLRP3 inflammasome after OGD/R in primary neurons. Primary neuronal cultures were prepared from E16-E18 C57BL/6 mouse brains. Briefly, meninges-free cortices were isolated, digested in trypsin-EDTA (0.1%) for 20 min, and dispersed in serum-free Neurobasal medium with 2% B27, 2 mM L-glutamine and penicillin/streptomycin for 7 d (cat. no.21103049, 17504044, 35050061, Life Technologies, Grand Island, NY). The cells were plated onto poly-L-lysine coated dishes (cat. no.801001, NEST) or 100-mm dishes at a density of 3–10×10^5 cells/mL. The cultured neurons were used for *in vitro* experiment and received different treatments as described above. Protein expression of TXNIP and NLRP3 was detected by Western blotting(A-C). Representative blots from six independent experiments with similar results are shown. Data are shown as mean ± SEM; * $P < 0.01$ *vs* Ctrl group, $^{\#}P < 0.05$ *vs* OGD group.
